# Traumatic rupture of the coronary sinus following blunt chest trauma: a case report

**DOI:** 10.1186/s13019-014-0164-y

**Published:** 2014-11-20

**Authors:** Do Wan Kim, Kyo Seon Lee, Kook Joo Na, Sang Gi Oh, Yong Hun Jung, In Seok Jeong

**Affiliations:** Department of Thoracic and Cardiovascular surgery, Chonnam National University Hospital, Chonnam National University Medical School, 42, Jebong-ro, Dong-gu, Gwangju, 501-757 South Korea; Department of Emergency Medicine, Chonnam National University Hospital, Chonnam National University Medical School, 42, Jebong-ro, Dong-gu, Gwangju, 501-757 South Korea

**Keywords:** Blunt trauma, Cardiac rupture, Coronary sinus, Extracorporeal membrane oxygenation

## Abstract

Cardiac rupture is rare but potentially life-threatening complication after chest trauma. We present the case of a 57-year-old male who developed cardiac arrest because of extensive pericardial tamponade after a falling injury. We decided to perform an exploratory sternotomy in the operating room (OR). The patient was transported to the OR on extracorporeal membrane oxygenation (ECMO) support. We found a rupture of the coronary sinus after evacuation of an extensive hematoma in the pericardium and primarily repaired the injured site. After 2 days, the patient died due to refractory cardiogenic shock. To our knowledge, this is the first reported case of rupture of the coronary sinus after blunt chest trauma.

**Electronic supplementary material:**

The online version of this article (doi:10.1186/s13019-014-0164-y) contains supplementary material, which is available to authorized users.

## Background

Patients with cardiac rupture, which is uncommon after blunt chest trauma, rarely survive and most die at the scene or soon in the emergency room before the cardiac lesions are disclosed [1]. The rupture of coronary sinus is usually associated with catheter-related complication of during open heart surgery [2]. However we were unable to find a previous report of traumatic rupture in coronary sinus after blunt chest trauma. Although there is no general agreement regarding its favorable effect in trauma fields, ECMO is expected to provide hemodynamic support in cases of refractory cardiac shock or arrest unresponsive to conventional resuscitation and to allow time for maintenance and recovery for further treatment [3]. We describe our unique experience of a case involving tearing of the coronary sinus following blunt chest trauma.

## Case presentation

A 57-year-old male visited the emergency room (ER) of our hospital after a fall injury from a height of ~3 m. At the time of admission, his vital signs were blood pressure (BP) 80/50 mmHg, heart rate 120 beat/min, and a respiratory rate increased to 26 breaths/min. Due to a decreasing level of consciousness and loss of voluntary respiration, we intubated the patient and estimated the small amount of pericardial effusion in Focused Assessment with Sonography for Trauma (FAST), and this finding did not deteriorate the hemodynamic condition. And then, we decided to perform the computed tomography (CT) scan for a more in-depth examination.

**Figure 1:**
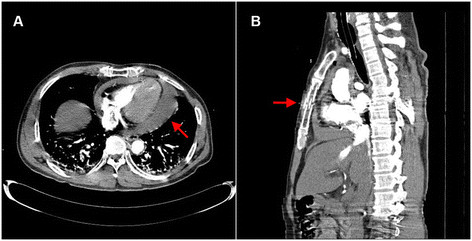
**Chest computed tomography at admission. (A)** The hemopericardium (red arrow) in the transverse view. **(B)** The sternal fracture (red arrow) in the saggital view.

However, the patient developed cardiac arrest during the CT scans. We performed cardiac massage for 20 min, but the heartbeat did not recover spontaneously. We inserted a cannula into his right femoral vein and femoral artery to start venous-arterial extracorporeal membrane oxygenation (VA-ECMO). After ECMO was started, his vital signs changed to blood pressure 120/68 mmHg, heart rate 143 beat/min, arterial blood gas analysis (ABGA) pH 7.24, partial pressure of CO_2_ 35 mmHg, partial pressure of O_2_ 419 mmHg, base excess (BE-E) -11.3 mmol/L, all of which improved since initiation of ECMO support. The chest CT results indicated cardiac tamponade with a large hemopericardium and sternal fracture. We diagnosed the patient with cardiac injury caused by the blunt trauma and decided to perform an emergency operation (Figure 1).

**Figure 2:**
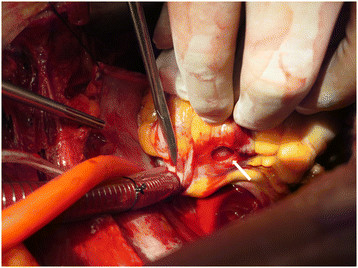
**An intraoperative image.** White arrow demonstrating a rupture in the distal one-third portion of the coronary sinus: an oval-shaped defect, 2 cm in its longest dimension, with sharp lacerations in the margin.

During a median sternotomy, we found a rupture in the distal one-third portion of the coronary sinus, an oval-shaped defect, ~2 cm in its longest dimension with sharp lacerations in the margin. There was an epicardial hematoma in the area surrounding the rupture, but no other damage was found. After we performed a conventional cardiopulmonary bypass (CPB), we discontinued the ECMO and attempted primary repair on the ruptured coronary sinus (Figure 2).

However, because we decided that the patient was not ready to be weaned off CPB, the ECMO flow was resumed and the patient was transferred to the ICU. There, we initiated therapeutic hypothermia while maintaining neuromonitoring using electroencephalography (EEG). On postoperative day 1, the patient was able to open his eyes spontaneously, but did not recover consciousness under sustained cardiogenic shock. On postoperative day 2, he died of refractory cardiogenic shock.

## Discussion

Blunt cardiac injuries occur infrequently, yet are dangerous and even life-threatening when accompanied by ruptures [1]. In this case, the patient developed rupture of the coronary sinus after blunt chest trauma. Although emergency surgery was performed under ECMO support, the patient eventually died.

According to autopsy reports, blunt cardiac injuries occur in 70% of all blunt chest trauma cases, and in most cases, the patient dies before arriving at the hospital [4]. Furthermore, autopsy studies on patients with trauma caused by fall injuries have reported blunt cardiac injuries in 54% of all cases [5]. In this case, because neurogenic shock, caused by the brain injury and cervical spine injury, seemed more likely at the time of admission, diagnosis of the cardiac injury was delayed. Accordingly, the patient developed cardiac arrest during his diagnosis and his condition was likely aggravated by the secondary injury caused by chest compression performed simultaneously. Additionally, in many cases with blunt chest trauma and sternum fracture, cardiac injuries are also common and the mortality rate is high [5]. As in this case, the trauma surgeon should keep in mind the possibility that a patient who has experienced a fall and has a sternum fracture is at high risk of developing cardiac injuries.

*In our center, we have a strategy that traumatic pericardial effusion must be explored by operative technique with CPB stand-by. To our knowledge, pericardiocentesis cannot be a definitive treatment in patients with traumatic tamponade. For the treatment of these patients, only surgical exploration should be chosen. Regarding the site of injury, right atrial injury is known to have best prognosis and on the other hand, the mortality of the patients with blunt chest trauma combined with cardiac rupture are reported to 85-100%. It will be expected that the survival can be improved by prehospital transport system and advanced life support.*

Although no multicenter randomized studies on ECMO for chest trauma have yet been published, ECMO has been gradually gaining attention as a treatment option in various trauma situations [3],[6]. In particular, ECMO is considered to be the treatment of choice in post-traumatic acute respiratory distress syndrome [6]. However, as most patients with trauma have a bleeding tendency, ECMO support also requires anticoagulation. The recent trend seems to show that ECMO runs without anticoagulation in traumatic patients, and they suggest that heparin-free ECMO support can improve outcome in initial stage [3]. In our case, although, ECMO only had the role of resuscitation procedure, it could not avoid or delay the high-dose anticoagulation during the definite cardiac surgery. Accordingly, ongoing advances in anticoagulation techniques and equipment are expected to increase the use of ECMO in patients with a high risk of bleeding [7]. Nonetheless, in cases in which hypovolemic shock or cardiac arrest has occurred after the development of cardiac rupture, more research is needed to determine whether ECMO can serve as a bridge to a definitive treatment, beyond a means of resuscitation [3].

## Conclusions

A case similar to ours has been reported, in which damage in the right atrium was prolonged and led to a coronary sinus avulsion [8]. However, based on our review of previous studies, no case in which an isolated injury in the coronary sinus occurred, as in the present case, has been reported. Although the patient did not survive, this case was unique and thus significant in terms of the way that intraoperative diagnosis of coronary sinus rupture was made. We also confirmed that early detection and treatment of the cardiac damage significantly affected the results of treatment.

## Consent

Written informed consent was obtained from the patient for publication of this case report and any accompanying images. A copy of the written consent is available for review by the Editor-in-Chief of this journal.

## Authors’ original submitted files for images

Below are the links to the authors’ original submitted files for images. Authors’ original file for figure 1 Authors’ original file for figure 2

## Abbreviations

OR: Operating room
ECMO: Extracorporeal membrane oxygenation
CT: Computed tomography
ABGA: Arterial blood gas analysis
EEG: Electroencephalography
CPB: Cardiopulmonary bypass
ER: Emergency room
