# Supplementary figures and images for: Traumatic rupture of the coronary sinus following blunt chest trauma: a case report
Source: J Cardiothorac Surg. 2014 Nov 20;9:164. doi: 10.1186/s13019-014-0164-y (PMC4246540; doi:10.1186/s13019-014-0164-y)

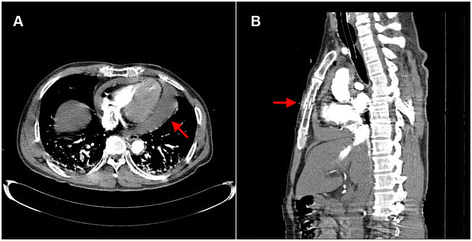

Supplement: Supplementary file 1 — Authors’ original file for figure 1 [file 13019_2014_164_MOESM1_ESM.gif]

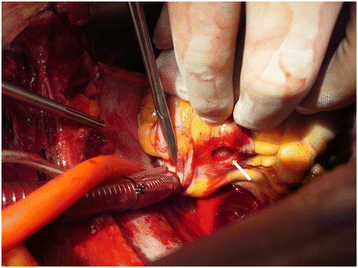

Supplement: Supplementary file 2 — Authors’ original file for figure 2 [file 13019_2014_164_MOESM2_ESM.gif]
